# “There’s Still Time to be Happy”: The Life Trajectories of Portuguese Transgender Women Who Transitioned at 50+ Years

**DOI:** 10.1177/23333936241236292

**Published:** 2024-04-18

**Authors:** Rita Carvalho, João Tavares, Tatiana Casado, Liliana Sousa, Sara Guerra

**Affiliations:** 1 University of Porto Institute of Biomedical Sciences Abel Salazar, Aveiro, Portugal; 2 University of Aveiro, Portugal; 3 University of the Balearic Islands, Palma de Mallorca, Illes Balears, Spain

**Keywords:** aging, gender identity, transgender, transitioning, LGBTIQA+, Portugal, envelhecimento, identidade de género, transgénero, transição, LGBTIQA+, Portugal

## Abstract

The process of transitioning involves making changes to align one’s life with their authentic gender identity. This study explores the life trajectories of three Portuguese transgender women who transitioned later in life (50+ years old) by identifying key chapters in their life courses. Through inductive thematic analysis, six chapters were identified from the participants’ interviews: (1) awareness of “something different in me,” (2) locked into suffering, (3) finding comfort in something that is socially recognized, (4) “it is enough”: it is time to recognize and embrace the woman I am, (5) living my life as a woman, and (6) building and leaving a legacy. Aging and the process of self-discovery played pivotal roles in participants’ process of transitioning. The perception of finitude and the limitations associated with the time of life led them to realize that there was no time to waste and a sense of urgency to live authentically.

## Resumo

O processo de transição envolve mudanças para alinhar a vida com a identidade de género autêntica. Este estudo explora as trajetórias de vida de três mulheres transgénero portuguesas que fizeram a transição mais tardia (depois dos 50 anos), identificando capítulos-chave nos seus percursos de vida. Através da análise temática indutiva, foram identificados seis capítulos a partir das entrevistas dos participantes: (1) consciência de “algo diferente em mim,” (2) fechada no sofrimento, (3) encontrar conforto em algo que é socialmente reconhecido, (4) “já chega:” é altura de reconhecer a mulher que sou, (5) viver a minha vida como mulher, e (6) construir e deixar um legado. O envelhecimento e o processo de autodescoberta desempenharam papéis fundamentais no processo de transição das participantes. A perceção da finitude e das limitações associadas ao tempo de vida levou-as a perceber que não havia tempo a perder, dando um sentido de urgência para viver de forma autêntica.

## Introduction

Transgender is an umbrella term used to designate those people who have a gender self-identity that is different from the biological sex assigned to them at birth (Cooper et al., 2020). For decades, transgender was considered a mental disorder. With the publication of DSM-III (American Psychiatric Association [APA], 1980) the diagnosis “transsexualism” first appeared. With the release of DSM-IV (APA, 1994), “transsexualism” was replaced with “gender identity disorder in adults and adolescence” in an effort to reduce stigma. With the publication of DSM-5 (APA, 2013), “gender identity disorder” was eliminated and replaced with “gender dysphoria.” The change further focused the diagnosis on the gender identity-related distress that some transgender people experience (and for which they may seek psychiatric, medical, and surgical treatments) rather than on transgender people or identities themselves. The DSM-5 articulates explicitly that gender non-conformity is not in itself a mental disorder (Cooper et al., 2020). In 2018, in the 11th version of the World Health Organization’s International Statistical Classification of Diseases and Health Problems (WHO, 2019), transsexuality was removed from the “Mental and Behavioral Disorders” chapter and was reclassified as “gender incongruence,” as part of the chapter “Conditions Related to Sexual Health.” This means that transgender people who are currently 50+ years old were confronted for decades with their identity being diagnosed as a mental disorder. The prevailing stigma surrounding both transgender identity and mental disorders significantly influenced the decision for many transgender people to remain closeted, fearing the potential consequences of disclosing their gender identity (White Hughto et al., 2015).

In Portugal, those who are now 50+ years old were born and raised under the grip of a dictatorship (Estado Novo) that lasted until 1974. It discouraged expressions of non-heteronormative sexuality, and LGBTIQA+ people could face arrest and imprisonment. “Transgressors” were destined for stigma and marginalization (Neves et al., 2023). Being transgender was classified as abnormal and sinful (Moleiro & Pinto, 2015). Living in such a society led transgender people experience internalized stigma; they tended to anticipate social rejection and believed that they were devalued members of society (Tsai et al., 2021). Consequently, these persons lived the first decades of their lives in an ambiance of stigma, oppression, and discrimination that led them to feel disrespected and invisible.

Since its transition to democracy (1974), Portugal has made significant progress in protecting the rights of all citizens, regardless of sexual orientation and gender identity. Two major laws protect the right to gender identity and recognize the self-determination of transgender citizens: Gender Identity (Law nº7/2011, of 15 March) and Gender Self-Determination (Law nº 38/2018, of August 7). The 2018 law streamlined the process of changing one’s gender identity and legal name, eliminating the requirement for a diagnosis of gender dysphoria, and allowing individuals to submit their requests to any civil registry office. These laws apply to people ≥18 years of age and of Portuguese nationality, whether living in Portugal or abroad. People aged between 16 and 18 years need parental consent accompanied by a non-pathologizing medical declaration attesting the free and informed will of the minor. Currently, in the ILGA Europe (2022) ranking, which positions countries on a scale between 0% (gross violations of human rights, discrimination) and 100% (respect for human rights, full equality), Portugal (with 62%) is in ninth position among EU countries and in ninth position, considering all countries.

Despite legal progress, transgender people in Portugal continue to face violence and discrimination (Neves et al., 2023). Although scarce, evidence suggests that transgender people are confronted with multiple forms of oppression in different contexts throughout their life span, indicating a global trend of trans-antagonism. From family to workplace, their fundamental rights are diminished or denied, with hate acts affecting personal, social, and professional relationships, even though such acts are legally prohibited (Neves et al., 2023). This marginalization is particularly acute among transgender older adults, who are more susceptible to stigma and oppression (Fredriksen-Goldsen et al., 2014). Studies have revealed that transgender older adults often age in isolation with limited access to supportive providers (Auldrige et al., 2012; Pinheiro et al., 2024). Further exacerbating the situation, a study by Fredriksen-Goldsen et al. (2014) revealed that transgender older adults exhibited significantly poorer physical health, disability, depressive symptomatology, and perceived stress compared to non-transgender LGB older adults. Hash and Rogers (2013) acknowledge “while these difficult experiences can create a host of problems for LGBT individuals, they can also help them develop unique skill sets or strengths that their non-LGBT counterparts do not necessarily benefit from as they age” (p. 249). Despite these challenges, some transgender people have successfully developed strong social networks, a positive sense of self, and ego integrity (Hash & Rogers, 2013).

While the bulk of existing research on transgender people primarily focuses on the challenges they face in various domains of life (e.g., mental health, gender-affirming hormone therapy, health-harming behaviors, HIV/AIDS, gender-affirming medical interventions and discrimination in healthcare settings) (Cicero et al., 2020), particularly during adolescence, it is equally important to recognize the transformative journey of transitioning. The process of transitioning involves making changes to be align one’s life with their affirmed gender identity. It is an individual and complex process that may vary in duration and trajectory, not always following a linear path. This process has been broadly characterized as encompassing three main changes (Campbell et al., 2019; Collazo et al., 2013; Evans et al., 2021): (a) social (“coming out” to friends and family, asking people to use a name and pronouns that match the new gender identity, dressing/grooming in ways that match your gender identity); (b) legal (changing the name on a birth certificate, driver’s license, or passport); (c) medical (the use of hormones and/or gender-affirming surgeries). Some transgender people need and desire transition in all three ways, while others do not (Austin & Goodman, 2017). Some of the literature suggests that transgender people may perceive transitioning as an ongoing process and one that never stops (a lifelong process); for some, it may involve achieving a desire level of physical congruence with their gender identity, while others may focus on social acceptance and recognition of their gender (Collazo et al., 2013; Morgan & Stevens, 2008). To understand the transitioning, the perspectives of transgender people need to be captured while acknowledging that pathways are individual and may vary widely (Evans et al., 2021; Schlittler et al., 2017).

The literature on the transitioning has focused on adolescents and young adults. Therefore, generational views are also important in understanding the experience of transitioning for the older transgender population (). However, some literature has focused on transitioning at 50+ years old, which normally occurs only after years, or decades, of significant frustration and of trying to “make things work” as cisgender (Fabbre, 2015). The literature has unveiled factors that lead transgender people to make or contemplate the transitioning at 50+ years old (Fabbre, 2015; Witten & Eyler, 2012). Major factors relate to better legislation, more supportive society, friends and families, and the availability of better information. There are individual factors, particularly the awareness of there being less time left to live and to embrace one’s authentic self (Cook-Daniels, 2016). Individuals have to ponder some pragmatic decisions: for instance, they may decide to wait until retirement before beginning the process of transitioning because they fear losing their jobs (Jones, 2020; Phoenix & Ghul, 2016). Family factors must also be considered, since many transgender people who come out later in life are in a heterosexual marriage and have children (Ashmore & Collier, 2017). Transitioning may lead to the end of a spousal relationship and the loss of custody of minor children because of gender role changes (Ashmore & Collier, 2017). Findings from a study by von Doussa et al. (2017)von Doussa et al. (2020) suggest that transgender people may highly value and protect their relationships with their families of origin (mostly their parents) or with the families they have created while living in their assigned gender (as partners, parents, siblings). This is often to the detriment of their gender expression, and they may delay their transitioning to protect their family relationships (von Doussa et al., 2020). Challenges also exist when seeking or retaining employment during transitioning (Jones, 2020).

For many transgender people, transitioning is a relief. However, when they are not supported, individuals may be at a high risk for suffering from discrimination, family rejection, anxiety, depression, violence, and suicidal attempts (Addis et al., 2009; James et al., 2016). Overall, research is needed to create a better understanding of the process of transitioning, particularly in those who transition at 50+ years old, by considering the transgender people’s perspective and inculcating cultural and social sensitivity (Brumbaugh-Johnson & Hull, 2019; Evans et al., 2021). In a society where gender norms have been rigidly defined, the experiences of transgender people who transition later in life offer a unique perspective on the complexities of gender identity and its intersection with age. Given the current climate of trans-antagonism, understanding their experiences is crucial because they may face additional challenges due to their age and the specific context of their transition. This study aims to explore the life trajectories of three Portuguese transgender women who transitioned at 50+ years old, identifying the main chapters of their life courses. In particular, we investigated the life trajectory that led them to transitioning at 50+ years old, and their life paths subsequent to their decision to transition.

## Methods

This is an exploratory-descriptive qualitative study (Hunter et al., 2019), based on primary sources, drawn from a larger study entitled “*Generativity, intended legacies, social participation and life satisfaction in LGBT+ older adults.”* That study aimed to contribute to a developmental perspective on the aging process among LGBTIQA+ people (Casado et al., 2023; Tavares et al., 2023). In this study, the data were analyzed using inductive thematic analysis (Braun & Clarke, 2006, 2021). Ethical approval was granted by the Ethics Committee of the Research Unit in Health Sciences of the Coimbra School of Nursing (UICISA: *E*-Ref. 714–10/2020). Presented here is a sub-corpus of the data, relating to the life trajectories of three transgender Portuguese women transitioning at 50+ years old.

### Recruitment

Recruitment for the main project started with contacts with LGBTIQA+ organizations to disseminate the study by sharing an online survey on generativity, intended legacies and satisfaction with life. The inclusion criteria were: (a) self-identification as LGBTIQA+, (b) aged ≥50 years old, (c) resident in Portugal, and (d) consent to participate. At the end of the survey, participants were asked about their willingness to collaborate in a semi-structured interview that included questions about life trajectories, generativity and intended legacies. Those willing to participate filled out a second form (independent of the first survey to ensure anonymity), indicating a phone number and/or e-mail address to be contacted by the research team. Between October 2020 and January 2021, 24 individuals completed the online survey: four self-identified as transgender (three women, one man). João Tavares and Sara Guerra contacted these participants by phone or e-mail to explain the study in greater detail, to confirm whether the participants met the inclusion criteria and to ascertain whether they remained interested in collaborating. The three self-identified as female or transgender women agreed to collaborate. The interview (online) was scheduled according to the participants’ convenience, and informed consent was obtained before starting. This article focuses on the three transgender women: their interviews took place in February and March 2021, and João Tavares and Sara Guerra conducted the interviews via Zoom in the Portuguese language. The interviews lasted, on average, 148.33 min (ranging from 40–240 min; *SD* = 82.50); two of the interviews were conducted on two occasions (2 different days), since the participants were prepared to share a lot about their lives.

### Participants

This study includes three Portuguese transgender women, aged between 56 and 65 years, who mentioned that their process of transitioning occurred at 50+ years of age (Table 1; names are changed for anonymity). Two were unemployed and one retired. Two were divorced from heterosexual marriages and had children. All three participants had made social transitions, including publicly, while the legal and medical transitions were something they still desired and were in progress.

**Table 1.** Participants.

**Beatriz** is 56 years old; she started her transition at 52 years of age. She is currently unemployed. She is divorced from a hetero marriage and has adult children.
**Maria** is 65 years old; she started her transition at 64 years of age. She is retired. She is divorced from a hetero marriage and has adult children.
**Liliana** is 58 years old; she started her transition at 56 years of age. She is currently unemployed. She has no children.

*Note.* *Some information was omitted to protect participants anonymity.

### Instrument

The semi-structured interview protocol comprised questions on life story (based on McAdams, 1985), generativity (McAdams & de St. Aubin, 1992), intended legacies (Newton et al., 2020), and sociodemographic data (age, gender, marital status, level of education, parental status, and professional status). The guiding questions were: (1) Life story: Can you briefly describe the trajectory of your life (the main chapters)? Are there any other important events that you would like to share? (2) Generativity: Have you an intention to contribute to future and present generations? What have you done or what do you plan to do? (3) Intended legacies: What do you expect to accomplish in the future (hopes, plans and dreams)? The interviews were recorded, transcribed and stored in a Word file. Rita Carvalho listened to the full records and proofread all the interviews to check for mistakes or inconsistencies. Notes and case summaries were written for each interview to record contextual observations and emerging ideas on important topics. These were shared between the interviewers and later, with the other members of the research team (the authors).

### Data Analysis

This study employed inductive thematic analysis (Braun & Clarke, 2006, 2021). Thematic analysis is an appropriate methodology for the research question (to describe the life trajectories of three Portuguese transgender women who transitioned at 50+ years old, by identifying the main chapters over their life courses), because it is a good method for examining the perspectives of different participants, highlighting similarities and differences (Braun & Clarke, 2006, 2021). The analysis was performed in European Portuguese. The quotes in this paper were then translated into English. Data analysis was performed in seven stages. First, Rita Carvalho read and re-read the interview transcripts and notes to become immersed in the original data. Second, she started the initial coding by exploring the content in relation to the study objective. Third, she shared the work done with João Tavares and Sara Guerra, and the three coders proceeded to break the data down to identify the most relevant information and identify themes. Fourth, Rita Carvalho, João Tavares and Sara Guerra met to discuss the themes and to search for connections between them. Fifth, the coders discussed the adequacy of the themes in relation to the three cases in the study. Sixth, the three coders met and, through a process of successive refinement, found patterns across the cases. In addition, to take the interpretation to a deeper level and to enhance analytic rigor, the research team (all authors) met to discuss the themes and to deepen the analysis by using metaphors (usually based on quotes from the participants) and temporal referents.

The research team for this study comprises a diverse group of young and experienced researchers with expertise in psychology, social work, nursing, and gerontology. This composition brings together a wealth of knowledge, perspectives, and experiences that enrich our understanding of the research topic. The team includes members of the LGBTIQA+ community, as well as allies, some involved in social movements advocating for equality and rights for LGBTIQA+ individuals. All team members are cisgender (non-transgender) people. Having a team made of LGBTIQA+ members and allies has facilitated contacts with transgender women, as well as the participants willingness to share their story in a deepen way. We believe that our diverse backgrounds and experiences position us to conduct this research with sensitivity, and competence, ensuring that the findings accurately reflect the lived experiences of the participants. Furthermore, we shared the findings/themes with the participants to ensure that we correctly interpreted their life trajectories.

## Results

The results are presented in a narrative format, using quotes from the participants to support the data and to ensure the findings are bonded to the participants’ life histories. The data analysis pointed to six chapters that represented the three participants’ experiences over their life courses: (1) an awareness of “something different in me,” (2) being locked into suffering, (3) finding comfort in something that is socially recognized, (4) “It is enough: it is time to recognize and embrace the woman I am,” (5) living my life as a woman and (6) building and leaving a legacy. All the participants decided to transition in the last 2–10 years and highlighted the importance of the historical period in which they had lived their life trajectories: *“You have to see my story as being about someone who is from a much older generation”* (Maria).

### Awareness of ‘Something Different in Me’

The participants all reported an awareness of *“something different in me.”* This occurred at different ages: in childhood (Beatriz), in adolescence (Maria) and as a young adult (Liliana). For Beatriz and Liliana, the awareness came mostly from feeling good when wearing women’s clothes and shoes:

When my parents went out (. . .), I dressed as a woman. . . and I liked to walk around like that. (Liliana)

When I put on women’s shoes, I felt a shiver all over my body. . . I felt good. (. . .) This was irresistible. (Beatriz)

Maria started feeling something different at school during adolescence, mostly because “*I started hanging out with other boys*.” She perceived that she was attracted to the boys.

The three participants described being confused. Beatriz mentioned that she did not feel “normal:” *“I noticed there was something different in me. (. . .) I used to change shoes with my classmates at school and I felt good doing that, but I wasn’t sure why. (. . .) I think I felt bad because I felt like I wasn’t normal”* (Beatriz). Maria felt confused because she was afraid of being gay: *“This traumatized me a lot (. . .) I didn’t feel attracted to women. Maybe I felt for men, but at that time I was so afraid of it”* (Maria). Liliana stated that she understood early on that she was attracted to men.

Her confusion came because she realized that she felt like a woman, but at that time, there was no accessible information about transgender people. Maria and Beatriz lived this period of confusion mostly in secret: *“I started to do these shoes and clothing changes in secret*” (Beatriz), while Liliana self-identified as a gay man.

### Locked Into Suffering

The confusion and secrecy led them to be *“locked into suffering*,” a suffering that came from the inside but was reinforced by the outside. Beatriz and Maria did not know what was happening to them. Liliana, despite knowing that she was a woman, *“never had the courage to assume (that role) because of society, family, everything.”* On the “inside,” the participants all described feeling bad (*“I lived in a prison”;* Liliana). They all recounted instances of oppression and discrimination that had a profound impact on their mental health, leading to episodes of self-injury and suicidal ideation:

I tried to commit suicide (. . .) I had depression and was hospitalized. (Liliana)

I went into a very bad phase. . . of denial, of mutilation. . .. (Maria)

I despised what was between my legs. I wished “that thing” would disappear the next day. (Beatriz)

The outside reinforced the suffering. Beatriz stated that others perceived her as a strange person. Maria thought that others perceived her as a transgender woman and therefore bullied her; she also emphasized a lack of support from her family (they did not understand her behavior and way of being). Her father verbally and physically abused (beat) her because “*he wanted to make a man out of me*.” Liliana, in contrast, felt compelled to conceal her feminine expressions from her parents, revealing an internal conflict between her authentic self and the fear of parental rejection:

I was the victim of bullying at school because I was the strange person, the one who didn’t talk, who didn’t get along! (Beatriz)

As a teenager, and in the following years, I lived tormented by mockery and social pressure as a result of my tics and my way of being. (Maria)

My mother and my father were against it [transitioning]; (. . .) I have suffered a lot. I encounter more prejudice at home than out of the home. (Liliana)

### Finding Comfort in Something That is Socially Recognised

The period of suffering led them to try to find comfort in something that was socially recognized. They wanted to feel good, and they tried in different ways to prove their adequacy; this was usually achieved by conforming to (more) acceptable ways of being and living. Maria and Beatriz both had marriages with women that were very important to them. For both, being in a heterosexual relationship was “*proof and evidence*” for themselves and for others that they were not homosexual (described as *“that horrible thing”* by Maria). Liliana had a different pathway since she understood that she was attracted to men and that she felt like a woman. However, she resigned herself to something that was more “acceptable” than being a transgender woman and engaged in relationships with men: *“I felt I was in the wrong body. But I couldn’t come out.”*

### It is Enough: It is Time to Recognize and Embrace the Woman I Am!

All participants stated that, in their middle age, they arrived at a turning point. After suffering and trying to adapt to the norms as best they could, there came a moment when they understood that they were women and decided “*It is enough: it is time to recognize and embrace the woman I am.”* Their exposure to information about transgender experiences and their personal connections with transgender people contributed to a clearer understanding of their own identities. Beatriz stated that she read magazines with news about transgender people and “*loved to know about that.”* However, she never thought *‘that [she] was “that.”* She was in Facebook groups and started to make some deductions: *“I’m married; I have children. . . so I’m going to look for other people who are trans and may have had a similar path.”* Moreover, all three participants described how an intimate relationship had enabled them to understand who they were. They were all involved in intimate relationships in which they were treated as women. This made them feel good, and they finally understood that they were women and wanted to be who they truly were.

I had an epiphany! I had a girlfriend who made me realise who I am. We became intimate and I think she started to relate to me as if I were a woman. Therefore, all [intimate/sexual] stimulation was as if I were a woman. Suddenly, I had multiple orgasms with high intensity. (. . .) Until I reached the point where I started to know what it was! (Beatriz)

She was a lesbian and we were totally uninhibited. It was a “lesbian” relationship; I felt like a woman. (Maria)

I’ve had two relationships [with men]; the second went well. Because that person told me that I looked like a woman (. . .) I felt good with him! (Liliana)

After feeling good as expressing their true gender identity and finally understanding who they were, they decided that they had had enough of living without expressing their identity (as women). Maria “*had psychiatric and friends” help*’ and the support of her children and grandchildren. Beatriz decided to get psychological support: *“I had consultations in a place that supports LGBTIQA+ people; then I started to educate myself about hormone therapy and stuff, talking to trans people.”* She also had support from her friends and children. Beatriz made her “coming out” while she was still married. However, her wife *“did not know how to manage the situation”* and ended up leaving her. Liliana looked for support from her friends (her parents did not accept it): *“I told them [my friends] my whole story. (. . .) All of them told me, ‘We will support you the best we can’. They are the ones who give me strength to continue”* (Liliana).

### Living My Life as a Woman!

All participants wanted or decided to *“live life as a woman”: “I feel like a cis woman, and I want to live my life that way”* (Beatriz); *“I hope to achieve what I have always been and wanted to be: a woman!”* (Liliana); *“If I can have a body that adjusts to who I am [a woman] (. . .) and I am able, psychologically and mentally, to reason as a woman. . . and to see my grandchildren and the people I love treat me as the person I am. . . this is my short-term goal*” (Maria).

They had traveled long journeys to assume their roles as women; therefore, they now wished to live life to the fullest as the women they had always been. Currently, they are still in the process of change: they had disclosed their gender identity, but now, all three women felt the need to move forward with the medical and legal transitions as well. Currently, they are wanting the outside (how they look to others) to match the inside (being a woman): “*I’m seeing physical changes in my body [due to surgeries] that are relieving my dysphoria a lot. . . and it’s an extraordinary feeling. Reborn!”* (Maria). They also want to be legally recognized as women: “*I think I’ll feel far more satisfied when I have an official document [the citizen’s card] that proves what I already am. Because it will give me recognition*” (Maria). They want to find emotional stability and to feel happy, to live their lives with serenity:

I hope these surgeries I’m doing can help, because two of them are decreasing physical and visual male characteristics. (Maria)

Now, I feel happy, but of course, there are concerns. There are things yet to happen, like the surgeries. (Beatriz)

Maybe I was never happy. Now, I am happy. Because I see my body change, I see the effects and it’s a joy. (Liliana)

Moreover, the participants stated that, in others’ eyes, they are still mostly perceived as a member of their assigned sex at birth, and they want to look like the women they are on the inside: *“Despite the physical changes I am making, if I go out (beyond my neighborhood) I am ‘sir’. (. . .) It inhibits me a lot and it anguishes me, and I end up not going out much”* (Maria).

### Building and Leaving a Legacy

In gaining their emotional stability, our participants are building a legacy. All three participants want to contribute, to maintain an active role and to share their stories to encourage others. They want to support other transgender people by giving them information and emotional support, and they want to inspire change in society’s mindset. Beatriz is helping another transgender people and wants to help others through being a role model:

She’s young (. . .). I make myself available. (. . .) As I transitioned very late, I just wish all trans people could have their epiphany as early as possible, to enjoy their life to the fullest. (. . .) And since I am from an older generation, I can make younger people think: “Hey, if even that old woman did this. . . then I’m young. . ..” It’s a bit like a mission. (Beatriz)

Maria wants to help and support other older transgender people: *“I am available to encourage and support people who may be at the same stage of life. (. . .) I know my limitations, but I want to play a role. I want to be involved”* (Maria). Liliana wants to help end prejudice and hatred: *“I would like to help change mindsets. (. . .) To diminish prejudice and hatred. To let society know that trans people are the same as others. People sometimes say ‘Ah, there’s a problem’, but we have no problem*.” In addition, all these women want to leave tangible legacies, such as writing a book (Beatriz), sharing her experiences in transgender forums (Maria), and becoming involved in activism movements and demonstrations (Liliana). Participating in this study is part of these more tangible legacies and exemplifies the role they want to play in society:

I’m 65 years old, (. . .) I want to play a role, I want to be involved. That was one of the reasons I responded to your survey and made myself available for this interview, because I think I can contribute in some way. (Maria)

## Discussion

The life trajectories of these three Portuguese transgender women reveal a complex and nuanced picture of the challenges and triumphs of transitioning later in life. These women all experienced significant oppression, violence, and discrimination throughout their lives. They faced bullying and harassment from peers, and pressure from family, and even threats of violence. Despite these challenges, they all found the courage to transition and live their lives authentically. The discrimination and oppression that they have experienced may have been a catalyst for social change, which is consistent with the empowerment theory (Gutierrez, 1994). They reported feeling happier and more fulfilled than ever before. Their stories are a testament of their resilience and show that it is possible to overcome adversity and live a full and meaningful live.

### Life Trajectory Chapters

Our results point to six chapters in the participants’ life trajectories. Overall, the six chapters may be grouped into two main periods of life: (a) living in a gender incongruent role (with the awareness that there is “something different in me,” being locked into suffering and finding comfort in something i.e., socially recognized) and (b) transitioning to live as the woman they are (“It is enough: it is time to recognize and embrace the woman I am,” living life as a woman, and building and leaving a legacy).

#### Living in a Gender Incongruent Role

Living in a gender incongruent role is a period that integrates the first three chapters: “Awareness that there is something different in me,” “Locked into suffering” and “Finding comfort in something that is socially recognized.” In this period, the participants were aware that they were different from others without understanding what it was they were experiencing. They felt confusion and fear, and, because of internal and external stigma, they were locked into suffering, going through depression, suicide attempts, denial and self-mutilation. They felt lack of support and had episodes of social isolation and depression, which is consistent with other studies (e.g., Grossman & D’Augelli, 2006; Nemoto et al., 2011; Simons et al., 2013).

Then, they found comfort in participating in something that was known and more socially accepted. The societal normalization of heterosexuality and gender conformity led our participants to follow a “natural” order (Fabbre, 2015; Orel & Fruhauf, 2013). Maria and Beatriz felt they needed to follow the heteronormative culture and they engaged in heterosexual marriages. Liliana believed that being a gay man was more acceptable than being transgender. These relationships were undertaken to reduce suffering and to mitigate the internal and external stigma. Current older transgender adults have denied or suppressed their feelings, sometimes for decades, in an attempt to fit into the roles that society expects of them (Cook-Daniels, 2016). Our participants tried to conform, but at a certain point they fail. However, as argued by queer theorists, there is liberation in a “failure” to conform (Fabbre, 2015; Jagose, 1996). And the liberation came with the decision to embark in the transitioning process.

#### Transitioning to Live as the Women They Are

After living in a gender incongruent role and trying to conform in different ways to feel good about themselves and to be accepted by society, a turning point brought each participant to a decision to transitioning. The chapter “It is enough: time to accept the woman I am” represents the turning point, a break with the suffering. This occurred due to contextual (greater information and contact with transgender people) and individual/relational factors (an intimate relationship in which they were treated as a woman and realizing that made them feel happy or good) (Fredriksen-Goldsen et al., 2013). In a life course perspective, identity development is embedded both by social changes and maturational factors (Floyd & Bakeman, 2006), which can happen at different stages of the life cycle (Cook-Daniels, 2016). Although this was not emphasized by our participants, the desire to protect their families (mainly parents, partners and children) can lead transgender people to slow down or pause their transitioning (von Doussa et al., 2020). Our participants had decided to transition in the last 2 to 10 years and were currently “living my life as a woman” and “building and planning to leave a legacy.” All the participants had made their social transitioning by presenting their real selves to relatives, friends and the public as transgender women. Perceived support in social disclosure has been associated with health, well-being, and satisfaction with life (Casado et al., 2023; Lewis et al., 2023). They were starting to live as woman in society, despite still being mostly perceived as a member of their assigned sex at birth and undergoing legal and medical transitions. After transitioning, the participants’ primary goal was to simply live as women in society (Ashmore & Collier, 2017). They wanted to pass as cisgender women, meaning they wanted the outside to match the inside (Cooper et al., 2020). Thus, they were going through medical transitions that are relevant but demand time and money. In Portugal, the NHS has just one specialized health unit (Genito-Urinary and Sexual Reconstructive Unit [URGUS]), created in 2011 to accompany individuals in the sex reassignment process, but the unit is unable to respond to all the needs. Therefore, transgender people, many in vulnerable socioeconomic situations, may have to go to private health units or wait for a long time. Accessible and affordable transitioning resources for transgender women are key to promote them with better quality of life (Glynn et al., 2016). Furthermore, medical transition in older adults has additional complexities due to aging factors. For instance, surgery carries more risks, such as infections and other complications (Beckwith et al., 2017). While going through their process of transitioning, our participants wanted to build and leave a legacy, mainly to prevent others (both younger and older) from suffering as much and for so long as they had (Casado et al., 2023; Harley et al., 2016; Sousa et al., 2010). They also wanted to contribute to changes in society (Austin & Goodman, 2017) that will diminish prejudice and hatred toward transgender people. Probably transgender women may also have to face unique forms of stigma within the LGBTIQA+ community, since some literature has underlined that those spaces may fluctuate in terms of support toward transgender people (Lewis et al., 2023).

### Cohort Perspective on Transitioning at 50+ Years of Age

As a scoping review to map transgender studies in Portugal demonstrates, information on the heterogeneous experiences and diversity of all individuals who self-identify as transgender is still scarce (Moleiro et al., 2023). For our participants, there were certain factors that influenced their decisions to come out and to transitioning, namely having more information due to social media, the contact with other transgender people, and the professional support (psychological and psychiatric) usually provided by LGBTIQA+ organizations or by specialized services available through the National Health System (Floyd & Bakeman, 2006). Their life trajectories were intertwined with social transformations (e.g., increased visibility and representation of transgender people in media and culture, greater access to information and resources, legal and policy changes) that enabled the transgender people to understand what was going on with them and to feel safer about making the transitioning later in life. Coming out is an individual journey driven by maturational factors *as well as* social changes (Fabbre, 2014). Transitioning at 50+ years of age seemed, for our participants, to have been mostly a generation situation, where social awareness and changes came together with self-discovery and sense of maturity to go forward with the transition (Fabbre, 2014). Age appears to have been a key factor in the participants’ decision to start transition at 50+. It was mainly their perception/awareness of finitude and the limitations associated with their time of life (which heightened the significance of experiencing authentic gender identity before they die) that led them to say, “Enough of not being *who I am!”* (Cook-Daniels, 2016; Fabbre, 2014). They felt that there was still time to be who they were, and they still had time to live life feeling good. This process was probably supported by the maturity and resilience acquired over many years of suffering (Austin & Goodman, 2017; Fredriksen-Goldsen et al., 2014; Patrão et al., 2019).

### Implications for Health Professionals

This study offers important implications for the training of health professionals, namely: (a) they can better understand the life trajectories of transgender women, acknowledging their resiliencies and challenges throughout life, which is essential for providing culturally competent and affirming care; (b) it can help health professionals to be aware of the importance of creating inclusive and safe environments, probably by involving transgender women who are willing to leave a legacy and contributing to others; and (c) it can help these professionals to develop/implement interventions that are specifically tailored to the needs of older transgender women (by addressing their specific health concerns, which can include hormone therapy, gender-affirming surgery, and mental health support). By incorporating these insights, healthcare professionals can play a crucial role in improving the health and well-being of transgender older adults.

## Limitations

Our study has several limitations. We had three participants, all living in urban areas and with secondary or higher levels of schooling. The sample is small, even though this is an exploratory study, but this community is difficult to reach. Further research could aim to reach transgender people living in rural areas and with lower levels of formal education. Furthermore, this study did not collect data on the ethnic, cultural, and religious backgrounds of the participants. This information could have provided valuable insights into the potential influence of these factors on the “coming out” experiences of these transgender woman. Future research should strive to include a more diverse sample in terms of ethnic, cultural, and religious backgrounds to gain a more comprehensive understanding of the unique challenges and experiences faced by transgender people from different communities. Other approaches to recruitment could also be used to reach a wider pool of potential participants, such as social media platforms. Additionally, we propose collaborating with LGBTIQA+ organizations to act as intermediaries in establishing contact with transgender people who come out later in life. Once initial connections are made, we could employ the snowball technique. Moreover, it would be relevant to address the life trajectories of transgender men transitioning at 50+ years of age. There are also transgender people who, although aware of their gender identity, decide not to transition or to do it just within a small group of close friends. Further research could also investigate their life trajectories with a view to portraying the blocking factors they encounter.

## Conclusion

There is a gap in transgender aging research and its specific and unique challenges, such as studies that address the multiple levels and intersecting influences (social, behavioral, psychological, and biological factors) over the life course (Moleiro et al., 2023). For our participants, transitioning at 50+ years seems to be leveraged by discovering the happiness of being who they really are (women), and it is possibly prompted by the pressure of the limited amount of time left to them. At around 50 years old, there is the perception that there is still time to be happy. Despite the challenging and threatening context of our participants’ earlier lives (e.g., prejudice and stigma), they display a marked resilience to the minority stress they experienced in their lives. A sense of resilience brings strength to the aging process. In fact, research has demonstrated that older LGBTIQA+ people report higher levels of life satisfaction and lower self-criticism (Barranti & Cohen, 2000). While our participants had done their “coming out,” many others from the same generation may still be suffering in isolation and trying to conform. While recognizing their transgender identities earlier might have alleviated some of the hardships they faced, our participants were not prepared to confront this aspect to their selves due to the significant familial, social, and legal barriers they encountered. Nevertheless, both aging and the process of self-discovery played crucial roles in their decision to transition later in life. The perception of finitude led them to realize that there was no time to waste. They had been women “since forever,” and they felt there was still time to be happy.
